# Features of Occupational Health Risks in the Russian Arctic (on the Example of Nenets Autonomous Okrug and Chukotka Autonomous Okrug)

**DOI:** 10.3390/ijerph18031061

**Published:** 2021-01-25

**Authors:** Sergei Gorbanev, Sergei Syurin, Aleksandr Kovshov

**Affiliations:** 1 Northwest Public Health Research Center, 191036 St. Petersburg, Russia; gorbanev@s-znc.ru; 2 Department of Hygiene of Educational, Training, Labor Conditions and Radiation Hygiene, Faculty of Preventive Medicine, North-Western State Medical University named after I.I. Mechnikov, 191015 St. Petersburg, Russia

**Keywords:** working conditions, occupational pathology, shortage of labor resources, Nenets Autonomous Okrug, Chukotka Autonomous Okrug, Russian Arctic

## Abstract

Working in the Arctic increases the risk of occupational diseases, which is especially important in the context of acute shortage of manpower in the region. The purpose of the study was to comparatively evaluate the working conditions and occupational pathology in Nenets Autonomous Okrug (NAO) and Chukotka Autonomous Okrug (ChAO) of Russia. We analyzed the results of socio-hygienic monitoring “Working Conditions and Occupational Morbidity” in 2008–2018. Despite similar climatic and socio-economic conditions, significant differences exist in the health risks of the working populations of the two regions. In NAO two-thirds of workers were employed at facilities with satisfactory sanitary and epidemiological well-being, while in ChAO only 13% of workers had such conditions. In NAO, almost all occupational diseases (93.2%) were due to exposure to noise among civil aviation workers. In ChAO, health problems mainly occurred among miners (81.5%). The most common of these were noise effects on the inner ear (35.2%), chronic bronchitis (23.1%), and mono- and polyneuropathies (12.5%). In 2008–2018, the occupational pathology risk in ChAO was higher than in NAO: RR = 2.79; CI 2.09–3.71. Thus, specificity of technological processes and forms of labor organization create significant differences in health risks for workers. It is necessary to use modern mining equipment to decrease the occupational morbidity in ChAO. In NAO, this effect can be achieved by updating the fleet of civil aviation.

## 1. Introduction

The Russian Arctic (Decree of the President of the Russian Federation dated 2 May 2014 No. 296 (revised 5 March 2020)) is the main source of raw materials for the country in the short and long term. Therefore, a powerful industry has been created in the region, and the scale of economic activity significantly exceeds the level of other polar countries. One of the problems of the Russian Arctic is the shortage of labor resources due to the actions of several factors. Firstly, there is a small number of the indigenous population employed mainly in traditional types of economic activities (reindeer husbandry, fishing, and sea animal hunting). Secondly, there is an insufficient number of people who have come from other regions of the country with professional skills to work in mining and processing enterprises. In addition, a significant number of nonindigenous inhabitants of the Arctic left the region due to the economic difficulties of the 1990s and early 2000s. Currently, the Russian Arctic, which occupies about 18% of the country’s territory, is home to no more than 1.5% of its 145 million population.

**Figure 1:**
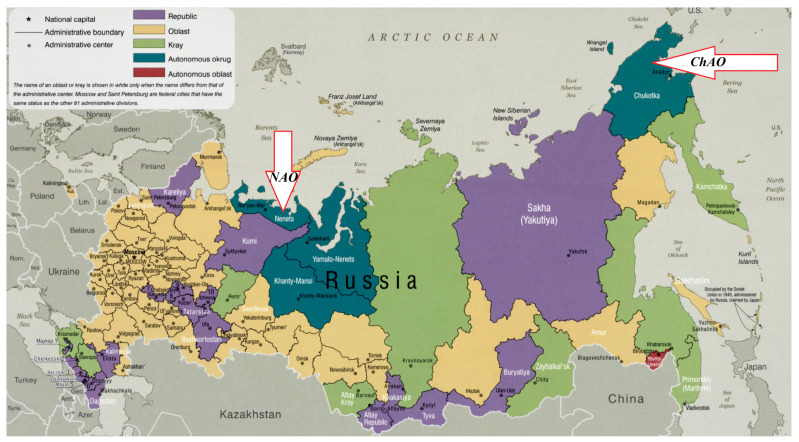
Political map of Russian Federation. (Available at: https://www.maps-of-the-world.ru/europe/russia/large-scale-administrative-divisions-map-of-russia-2009).

In full measure, the problem of a shortage of labor resources is relevant for the Nenets Autonomous Okrug (NAO) and Chukotka Autonomous Okrug (ChAO) (Figure 1), the populations of which are 44.1 and 50.7 thousand people, and the densities are 0.25 and 0.07 people/km^2^, respectively (as of 1 January 2020). In addition, with extreme climatic conditions, underdeveloped social and economic infrastructures are common to both regions. Their difference lies in the leading type of economic activity. In NAO, the economy is based on the extraction of oil and natural gas, whereas the extraction of hard and brown coal, alluvial and ore gold, and other nonferrous metal ores prevail in ChAO. Despite the noted difficulties in terms of the size of the gross regional product per capita in 2018, NAO ranked first (6288.5 thousand rubles) and ChAO ranked fifth (1.386.1 thousand rubles) in Russia [1,2].

It is known that the climatic conditions of the Arctic modify the action of harmful occupational factors, increasing the risk and reducing the time of formation of occupational diseases [3,4,5]. In 2008–2018, the level of occupational morbidity in the Arctic, in contrast to the Russian Federation as a whole, tended to increase [6,7]. Previous studies were mainly devoted to the problems of populous and economically more important regions of the Arctic: Murmansk oblast, Yamalo-Nenets Autonomous Okrug, and Krasnoyarsk Krai [4,5,8]. At the same time, due attention was not paid to the NAO and ChAO, which have less access to the leading scientific and medical centers of the country. It is important to note that the early termination of labor activities due to occupational diseases is an additional factor that aggravates the shortage of labor resources in the northern regions [9,10].

In Russia, monitoring of working conditions, occupational health, and morbidity falls within the competence of the Russian Federal Service for Surveillance on Consumer Rights Protection and Human Wellbeing (Rospotrebnadzor). All standards for working conditions, regulations for periodic medical examinations of workers exposed to harmful occupational factors, criteria for occupational diseases, and their list and registration rules are the same for the whole country and are regulated by Federal laws and orders of the Ministry of Health of Russia. Only teams of doctors specially created for this purpose, trained in occupational safety and health, have the right to diagnose an occupational disease. Data on working conditions and indicators of occupational morbidity in all 85 regions of Russia and at the federal level are updated annually and presented on the Internet in the form of government reports.

Taking into account the especially difficult demographic situation in the northern regions of the country [11], studying the influence of harmful environmental factors, including the working environment, and creating a set of measures for maintaining the health of the working population are two of the priorities of the state policy of the Russian Federation in the Arctic (On the fundamentals of the state policy of the Russian Federation in the Arctic for the period until 2020 and beyond. Rossiyskaya Gazeta, No. 4877 (18 September 2008)).

The aim of this research was a comparative study of causes, structures, and incidence of occupational pathology in the two Russian Arctic regions: Nenets Autonomous Okrug and Chukotka Autonomous Okrug.

## 2. Materials and Methods

The study used information and data from the Russian Federal Information Fund for Social and Hygienic monitoring (section “Working conditions and occupational disease incidence”) in NAO and in ChAO in 2008–2018 (Access to the materials is provided only to public health professionals. However, part of the information is published annually in state reports on sanitary and epidemiological well-being in Russia. Available at: https://rospn.gov.ru/documents/documents.php (accessed: 2021/01/06). (in Russian)).

The analysis included data on the annual number of employees of enterprises who had contact with ambient factors in workplaces (hazardous substances, fibrogenic aerosols, electric and magnetic fields, heat and cold, noise, vibration) as well as information about heavy physical work and psychosocial hazards and risks (working arrangements, routine work, intellectual, sensory, and emotional stress). We determined the number of jobs in enterprises with satisfactory, unsatisfactory, and extremely unsatisfactory working conditions (classified according to the data of a comprehensive assessment of the action of all factors of the production environment and the labor process). The analysis also dealt with all newly registered cases of health disorders of occupational etiology (diseases, intoxications, and their nosological forms, acute or chronic course of the process). In addition, we assessed data on gender, age, profession, length of service of sick persons, the nature of harmful production factors, and the exposure circumstances, the circumstances of the initial detection of health disorders of occupational etiology.

Microsoft Excel 2010 and IBM SPSS Statistics v. 22 programs were used to process the study findings. We used a Kolmogorov–Smirnov test to determine the normal distribution in the “Age” and “Employment duration” samples in NAO and ChAO, a Mann–Whitney criterion (U-test) (also for these samples), agreement criterion χ^2^ (for unpaired nominal data, i.e., to compare NAO and ChAO) or Fisher exact (one-tailed) criterion (if the number of observations in the sample was less than 5), and McNemar’s test (for paired nominal data, i.e., to compare indicators in one of the territories in 2018 and 2008). We used the polynomial trend (*n* = 4) to assess the dynamics of occupational morbidity indicators, the forecast for 1 year was given, and the coefficient of determination of the model (R^2^) was calculated.

We calculated relative risk (RR) and 95% confidence interval (CI). RR was calculated as the incidence rate of the outcome in the tested group, divided by the outcome of the control group. CI was determined by using a two-step procedure: CI was generated for Ln(RR), and then the antilogs of the upper and lower limits of CI for Ln(RR) were computed to give the upper and lower limits of Cl for the RR [12].

Numerical data are presented as absolute values, percentages, and a mean with a standard deviation or a median with the interquartile range (IQR, Q_1_, and Q_3_). The critical level of significance for the null hypothesis was 0.01.

## 3. Results

The study showed that the most prevalent occupational hazards (percentage of each being over 10% of cases) to which NAO enterprise employees were exposed in 2008–2018 included nonionizing electromagnetic fields and radiations, cooling workplace microclimate, heavy physical work, noise, and chemical hazards. Working conditions changed insignificantly over the eleven years. A decreased percentage of workers exposed to noise and increased percentage of those exposed to chemical hazards (*p* < 0.001) in 2008 and 2018 were the only reported changes. The share of other occupational hazards in their total spectrum has not changed significantly.

Cooling workplace microclimate, noise, and the combined action of several occupational hazards were most prevalent at ChAO enterprises. Since 2008, the percentage of workers exposed to cooling workplace microclimates, heavy physical work, fibrogenic aerosols, and chemical hazards (*p* < 0.001) decreased in 2018. At the same time, the combined action of occupational hazards, nonionizing electromagnetic fields and radiations, and hand–arm vibration increased (*p* < 0.001). No changes in the prevalence of whole-body vibration or psychosocial hazards and risks were detected. According to the average annual indices in 2008–2018, at the enterprises of NAO, in comparison with ChAO, a greater share of employees was exposed to the cooling microclimate, heavy physical work, psychosocial hazards and risks, nonionizing electromagnetic fields and radiations, and chemical hazards (*p* < 0.001). A larger number of workers at ChAO enterprises as compared to NAO were exposed to a combined effect of occupational hazards, noise, whole-body and hand–arm vibrations, and fibrogenic aerosols. If we assess working conditions by the dynamics of particular occupational hazards throughout 2008–2018, an absence of significant changes in NAO and tendency to improve in ChAO can be noticed (Table 1).

A comprehensive assessment of working conditions based on the percentage of workers employed at the production facilities of three groups of sanitary-epidemiological well-being showed that in NAO in 2008–2018, almost two-thirds of employees worked in satisfactory working conditions (the first group). Over eleven years, the number of persons with satisfactory working conditions increased (*p* < 0.001), and the number of workers with unsatisfactory conditions (the second and third groups) decreased (*p* < 0.001). In 2018, there was no enterprise left in NAO, characterized by extremely unsatisfactory sanitary-epidemiological well-being (the third group). In 2008–2018, only 13% of workers in ChAO were employed at enterprises with satisfactory working conditions, and almost one third of workers were reported to have extremely unsatisfactory sanitary-epidemiological well-being. For eleven years, there has been an increase in the proportion of workers at the facilities of the first and second groups (satisfactory and unsatisfactory conditions), while at the facilities of the third group (extremely unsatisfactory conditions) it decreased. According to a complex assessment during 2008–2018, working conditions at the enterprises of NAO were more favorable than in ChAO, which was manifested by a larger share of workers employed at the facilities of the first group and a smaller share of workers engaged at the second and third group facilities (*p* < 0.001). It is also important that the improvement of working conditions that took place in 2008–2018 was more significant in NAO than in ChAO (Table 2).

In 2008–2018, 59 and 216 occupational diseases were detected for the first time in NAO and ChAO, respectively. Almost all cases were men. The seniority at the time the disease was established was higher among workers in NAO, who were employed in air transport and in oil and gas production. In ChAO the percentage of mining enterprise workers (coal, lignite, ore raw materials, and alluvial gold) was higher and the percentages of air transport employees was lower than in NAO. Of the 176 occupational diseases identified in miners in ChAO, 106 cases were related to the extraction of coal and lignite and another 70 cases were related to the extraction of ore raw materials (Table 3).

Almost all occupational diseases among NAO enterprise employees resulted from exposure to industrial noise and only two cases were related to heavy physical work. The formation of occupational pathology among the employees of enterprises in ChAO was caused by seven occupational hazards. The most common of them were noise and fibrogenic aerosols. Physical factors (noise, hand–arm and whole-body vibrations) predominated (55.1%) in the structure of harmful production factors that caused occupational diseases among employees in ChAO. In NAO, occupational pathology formation was in most cases (89.8%) due to equipment design defects. In ChAO, occupational diseases were associated with imperfection of technological processes and, to a lesser extent, with design flaws and malfunction of machines, mechanisms, equipment, devices, and tools (Table 4).

Occupational diseases in NAO workers included only two nosological forms: noise effects of inner ear and radiculopathy. Occupational pathology structure of ChAO workers was much more diverse. Among the diagnosed health problems were diseases of ear, musculoskeletal and nervous systems, injuries and other consequences of exposure to external causes, respiratory diseases, and malignant neoplasm. However, as with NAO workers, noise effects of the inner ear were the most prevalent health problem. Respiratory diseases (chronic bronchitis, pneumoconiosis) were detected mainly in coal and lignite miners. All occupational health disorders had a chronic course (Table 5).

Occupational pathology in NAO was first detected after mandatory periodical medical examinations (53 cases or 89.8%), and only six diseases (10.2%) were identified as a result of workers’ self-requests for medical help because of poor health. In ChAO, there was the opposite ratio of occupational diseases diagnosed during medical examinations and due to workers´ self-requests for medical assistance: 57 (26.4%) and 159 (73.6%) cases, respectively.

The annual number of occupational diseases first diagnosed in NAO ranged between 2 (in 2008 and 2011) and 9 (in 2018) cases. The level of occupational morbidity in the okrug did not significantly differ from the all-Russian one. The eleven-year curve of occupational disease rate in NAO had a “sawtooth” shape, with rises and falls, and there was a slight general upward trend. In 2018, the risk of occupational pathology development in NAO was not higher than in 2008: RR = 2.99; CI 0.65–13.8; χ^2^ = 2.17; *p* = 0.141.

**Figure 2:**
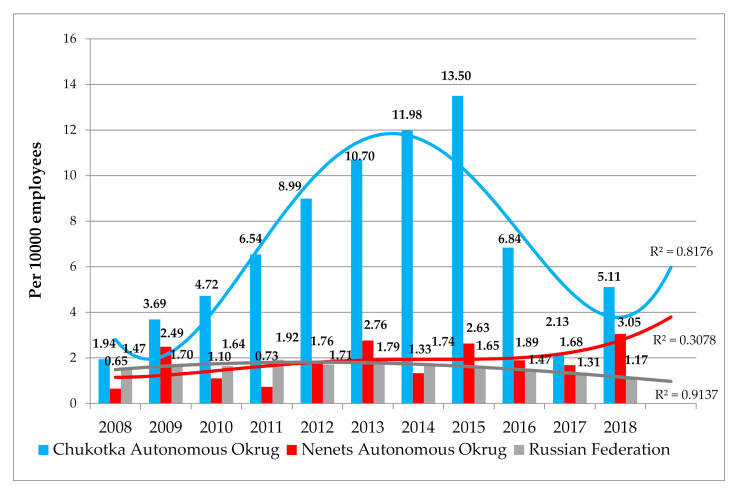
Occupational morbidity rates in Chukotka Autonomous Okrug, Nenets Autonomous Okrug, and Russian Federation in 2008–2018 (per 10,000 employees).

In ChAO, the annual number of newly diagnosed occupational diseases ranged from 6 (2008 and 2017) to 37 (2015), which caused significant changes in the level of occupational morbidity. In 2008–2015, it was growing steadily. In 2016–2017, there was a decrease in indicators, followed by their rise in 2018. Over eleven years, occupational disease level in ChAO exceeded the all-Russia indicators. In 2018, occupational pathology risk in ChAO significantly exceeded the level of 2008: RR = 3.46; CI 1.34–8.91; χ^2^ = 7.48; *p* = 0.006 (Figure 2). It was not possible to identify changes in working conditions with which the subsequent dynamics of occupational morbidity indicators could be associated, both in NAO and ChAO. In general, in 2008–2018, the risk of occupational pathology formation among employees of enterprises in ChAO was higher than in NAO: RR = 2.79; CI 2.09–3.71; χ^2^ = 53.6; *p* < 0.001.

## 4. Discussion

This study showed that, despite the similarities of climatic, demographic, and social indicators in NAO and ChAO, the working conditions, structure, and incidence of occupational pathology in these two Arctic regions have significant differences. In 2008–2018, more favorable working conditions were seen in NAO, as almost two-thirds of employees were engaged in facilities with satisfactory indicators of sanitary-epidemiological well-being. This can be also evidenced by a longer employment duration period before occupational pathology development as compared with workers at ChAO enterprises. The data obtained contradict the information on the increased risk of developing diseases of the musculoskeletal and nervous systems and circulatory and respiratory organs in oil and gas industry workers in the subarctic and arctic regions, summarized in the report of the International Labor Organization [13].

In NAO, the vast majority of occupational pathology cases were formed not in the oil and gas industry workers, as one might have expected, but among civilian flight personnel exposed to noise. In addition to more favorable working conditions, one of the possible explanations for rare cases of occupational pathology in oil and gas production may be the widespread use of the shift method of work. First, the detection and registration of diseases is known to be extremely difficult in shift workers [14,15]. Secondly, shift workers are people with initially better health indicators than the population of the region and the country as a whole [16]. One should also consider a tendency of some employees to hide the true state of their health in order to maintain highly paid jobs in the oil and gas industry in the Arctic [17,18]. In general, the data obtained are consistent with the level of occupational morbidity in Russia in the extraction of natural resources. In 2017, in oil and gas production, it amounted to 2.12 cases per 10,000 employees, and in the extraction of all types of raw materials—26.87 cases per 10,000 employees [7].

In 2008–2018, only 13% of ChAO workers were employed at facilities of the first group of sanitary-epidemiologic well-being, which explains the higher level of occupational disease rate and its tendency to grow over the past eleven years. Occupational pathology structure in ChAO is typical for mining enterprises. It includes musculoskeletal, nervous and respiratory system diseases, noise effects of the inner ear, injuries, and other external effect outcomes [19,20,21,22].

The dynamics of occupational morbidity in Chukotka is characterized by a pronounced rise from 2008 to 2015, a sharp decline in 2016–2017, and a subsequent upward trend from 2018. As we have already noted, we were unable to identify changes in working conditions that could cause such dynamics. Based on the data of the Russian Federal Information Fund, we can state a significant improvement in working conditions only at energy facilities. However, these objects initially made little contribution to occupational morbidity. We assume that the sharp decline in morbidity may be associated with a deterioration in the quality of medical examinations of employees. In 2017, 100% of cases of occupational diseases were detected when employees independently sought medical help, while in 2015 the share of such cases was 59.26%.

A single case of occupational pathology in women, while in Russia their number is 14.2–42.2% [23], is explained by a sharp restriction on the use of female labor in the mining industry and as pilots in the civil aviation. Only two cases of occupational pathology in extreme climatic conditions of NAO and ChAO were related to a cooling workplace microclimate. In this situation there was probably an incomplete account of the effect of exposure to cold on the health of workers. This fact may be due to the peculiarities of the special assessment of working conditions currently used in Russia. In ChAO, it is noteworthy that most occupational diseases were detected because of self-request of workers for help, which may be a consequence of the low quality of periodic medical examinations [24,25].

It is logical to compare the prevalence and structure of occupational pathology in NAO and especially in ChAO with similar indicators in the geographically close American state of Alaska. Problems of occupational pathology, as well as cases of insect and animal bites, various injuries and poisoning associated with industrial activities, are within the competence of the Alaska Occupational Disease and Injury Surveillance System. The literature provides data on work-related nonfatal health disorders, assessed by the number of compensation claims filed by injured workers [26,27] and by the number of accidents [28,29]. Therefore, in 2014–1015 there were 44 such requirements for all sectors of the economy per 1000 employees. The highest rates were observed for seafood processors (63 cases per 1000 employees). Less frequently, compensation was demanded by builders (34 per 1000), food processors (37 per 1000), car drivers (38 per 1000), medical workers (45 per 1000), and other professionals. Unlike ChAO and NAO, there were no miners, oil workers, or pilots among them. Among health disorders, injuries prevailed, which most often occurred among firefighters (162 cases per 1000 workers) and law enforcement officers (121 cases per 1000 workers) and less often among loggers, aviation personnel, and other workers.

The analyzed articles mainly discuss the causes and frequency of occupational injuries. Only among seafood processors have diseases of the musculoskeletal system, caused by physical overstrain and frequent repetitive movements, been described [26]. There are no data on diseases associated with exposure to industrial vibration, noise, and dust aerosols. However, it is these factors, along with heavy physical work, which cause the development of most cases of occupational pathology in NAO and ChAO. Unfortunately, we have to admit that differences in the interpretation and registration of occupational health disorders in Russia and the United States make it impossible to compare them in the Arctic regions of the two countries.

## 5. Conclusions

The technological specifics of the extraction of natural resources and the organization of work in the Arctic create significant differences in health risks for workers at enterprises in NAO and ChAO. In the Arctic region, there is a higher risk of occupational pathology in workers employed in the mining industry than in the extraction of oil and gas. To decrease the occupational morbidity rate in Chukotka miners, it is necessary, first of all, to improve technological equipment and technological processes, aimed at reducing levels of noise, whole-body, and hand–arm vibration, concentration of fibrogenic aerosols, and heavy physical work. A decrease in occupational morbidity rates in NAO can be achieved by updating the civil aviation fleet with aircraft with reduced noise characteristics and by the use of more effective individual antinoise equipment. Special attention should be paid to improving the quality of periodical medical examinations of industrial workers in ChAO.

## Figures and Tables

**Table 1:** Share of employees exposed to major occupational hazards (%).

Occupational Hazards	Year						Average Annual Indicator
	2008	2010	2012	2014	2016	2018	
Cooling microclimate	14.0 11.0	19.8 17.0	16.6 13.8	20.9 11.5	13.5 9.4	13.6 8.2	16.4 11.8
Heavy physical work	16.2 9.9	17.1 5.4	11.2 3.7	10.9 10.4	12.6 6.1	14.5 6.9	13.8 7.1
Noise	15.0 19.6	14.9 21.6	11.4 18.3	17.4 15.5	8.2 13.5	7.6 16.0	12.4 17.4
Non-ionizing electromagnetic fields and radiations	28.9 7.3	29.6 2.5	33.2 7.8	22.4 6.0	29.8 10.1	28.0 9.1	28.7 7.1
Chemical hazards	8.9 12.0	7.4 5.7	10.0 7.4	10.6 6.5	13.4 4.2	13.3 4.6	10.6 6.7
Psychosocial hazards and risks	8.2 2.5	3.3 2.0	3.1 3.4	4.1 3.3	5.7 2.7	8.3 2.4	5.5 2.7
Whole-body vibration	1.0 6.4	1.5 9.7	2.4 8.4	1.6 6.3	2.7 6.4	0.6 7.0	1.6 7.4
Fibrogenic aerosols	1.0 11.6	1.0 12.3	0.6 7.6	0.4 4.6	2.1 5.1	0.9 4.7	1.0 7.7
Hand–arm vibration	- 1.7	- 2.0	- 2.4	- 4.2	- 2.2	- 3.8	- 2.7
Combined effect of occupational hazards	8.3 12.5	4.0 15.9	9.9 19.7	8.4 15.4	9.0 32.8	8.4 28.4	8.0 20.8

Notes: upper line—indicators in Nenets Autonomous Okrug (NAO), lower line—indicators in Chukotka Autonomous Okrug (ChAO).

**Table 2:** Share of workers at enterprises of three groups of sanitary-epidemiological well-being (%).

Group of Enterprises (According to Sanitary and Epidemiological Well-Being)	Year						Average Annual Indicator
	2008	2010	2012	2014	2016	2018	
First (satisfactory)	48.3 12.0	56.5 10.7	62.3 14.8	62.0 14.1	61.8 12.1	83.8 14.5	62.5 13.0
Second (unsatisfactory)	43.9 47.7	38.5 52.0	33.2 49.8	32.5 50.5	35.4 58.0	16.2 55.3	33.3 52.2
Third (extremely unsatisfactory)	7.8 40.3	5.0 37.3	4.5 35.3	5.5 35.4	2.8 29.9	- 30.2	4.3 31.9

Notes: Upper line—indicators in NAO, lower line—indicators in ChAO.

**Table 3:** General characteristics of workers with newly diagnosed occupational diseases.

Indicator	Arctic Region		*p*
	NAO (*n* = 59)	ChAO (*n* = 216)	
Age, years (median, IQR)	57.0 [53.0–59.0]	55.0 [51.0–58.0]	0.014
Employment duration, years (median, IQR)	32.0 [29.0–36.0]	28.0 [22.3–33.0]	<0.001
Diseases in males, cases	59 (100.0%)	215 (99.5%)	0.786
Disease in females, cases	0	1 (0.5%)	
Extraction of all minerals (ore, coal, gas, lignite, oil), cases	4 (6.8%)	176 (81.5%)	<0.001
Air transport, cases	55 (93.2%)	38 (17.6%)	<0.001
Electric power generation and distribution, cases	0	2 (0.9%)	0.616

**Table 4:** Factors and circumstances associated with occupational disease development (cases and %).

Indicator	Occupational Diseases		*p*
	NAO (*n* = 59)	ChAO (*n* = 216)	
Factors:			
noise	57 (96.6%)	76 (35.2%)	<0.001
fibrogenic aerosols	0	68 (31.5%)	<0.001
hand–arm vibration	0	27 (12.5%)	<0.001
heavy physical work	2 (3.4%)	25 (11.6%)	0.043
whole-body vibration	0	16 (7.4%)	0.019
chemical hazards	0	2 (0.9%)	0.616
cooling microclimate	0	2 (0.9%)	0.616
Circumstances:			
imperfection of technological processes	6 (10.2%)	139 (64.4%)	<0.001
design defects of machines, mechanisms, equipment, tools, and instruments	53 (89.8%)	53 (24.5%)	<0.001
malfunction of machines, mechanisms, equipment, tools, and instruments	0	24 (11.1%)	0.002

**Table 5:** Clinical characteristics of occupational pathology (cases and percentages).

Occupational Pathology	Arctic Region		*p*
	NAO (*n* = 59)	ChAO (*n* = 216)	
Noise effects on inner ear	57 (96.6%)	76 (35.2%)	<0.001
Chronic bronchitis	0	50 (23.1%)	<0.001
Mono- and polyneuropathies	0	27 (12.5%)	<0.001
Pneumoconiosis	0	19 (8.8%)	0.009
Vibration disease	0	16 (7.4%)	0.019
Radiculopathy	2 (3.4%)	15 (6.9%)	0.315
Deforming osteoarthrosis	0	6 (2.8%)	0.231
Forearm myofibrosis	0	4 (1.9%)	0.378
Spinal osteochondrosis	0	2 (0.9%)	0.616
Pulmonary malignant neoplasm	0	1 (0.5%)	0.786

## Data Availability

Restrictions apply to the availability of the data presented in this study. Data was obtained from the Russian Federal Service for Surveillance on Consumer Rights Protection and Human Wellbeing (Rospotrebnadzor) and are available on request from the corresponding author (kovshov@s-znc) with the permission of Rospotrebnadzor.
